# The value of machine learning technology and artificial intelligence to enhance patient safety in spine surgery: a review

**DOI:** 10.1186/s13037-024-00393-0

**Published:** 2024-03-25

**Authors:** Fatemeh Arjmandnia, Ehsan Alimohammadi

**Affiliations:** 1 Department of Aneasthesiology, Kermanshah University of Medical Sciences, Kermanshah, Iran; 2 Department of Neurosurgery, Kermanshah University of Medical Sciences, Imam Reza Hospital, Kermanshah, Iran

**Keywords:** Machine learning techniques, Patient safety, Spine surgeries, Surgical procedures, Patient outcomes

## Abstract

Machine learning algorithms have the potential to significantly improve patient safety in spine surgeries by providing healthcare professionals with valuable insights and predictive analytics. These algorithms can analyze preoperative data, such as patient demographics, medical history, and imaging studies, to identify potential risk factors and predict postoperative complications. By leveraging machine learning, surgeons can make more informed decisions, personalize treatment plans, and optimize surgical techniques to minimize risks and enhance patient outcomes. Moreover, by harnessing the power of machine learning, healthcare providers can make data-driven decisions, personalize treatment plans, and optimize surgical interventions, ultimately enhancing the quality of care in spine surgery. The findings highlight the potential of integrating artificial intelligence in healthcare settings to mitigate risks and enhance patient safety in surgical practices. The integration of machine learning holds immense potential for enhancing patient safety in spine surgeries. By leveraging advanced algorithms and predictive analytics, healthcare providers can optimize surgical decision-making, mitigate risks, and personalize treatment strategies to improve outcomes and ensure the highest standard of care for patients undergoing spine procedures. As technology continues to evolve, the future of spine surgery lies in harnessing the power of machine learning to transform patient safety and revolutionize surgical practices. The present review article was designed to discuss the available literature in the field of machine learning techniques to enhance patient safety in spine surgery.

## Introduction

The integration of machine learning technologies in healthcare has revolutionized patient care and safety across various medical specialties [1–5]. In the context of spine surgeries, where precision and risk mitigation are paramount, the application of machine learning algorithms offers a promising avenue for improving patient outcomes and reducing complications [1, 6–10]. This article explores the potential of utilizing machine learning approaches to enhance patient safety in spine surgeries, aiming to optimize surgical decision-making, personalize treatment strategies, and ultimately elevate the standard of care in this critical field of medicine.

## Leveraging machine learning for enhanced patient safety in spine surgeries

Leveraging machine learning techniques for enhanced patient safety in spine surgeries represents a significant advancement in the field of healthcare, offering a data-driven approach to improving surgical outcomes, reducing complications, and optimizing care delivery in spine surgery practices. By harnessing the power of machine learning algorithms, predictive analytics, and real-time decision support tools, healthcare providers can leverage vast amounts of data to identify patterns, trends, and predictive factors that can help optimize surgical decision-making, personalize treatment plans, and enhance patient safety throughout the surgical process [5, 11, 12].

One of the key advantages of utilizing machine learning in spine surgeries is its ability to analyze and interpret complex datasets, such as patient demographics, medical history, imaging studies, and surgical parameters, to generate actionable insights and personalized recommendations for surgeons. By processing and synthesizing this data, machine learning algorithms can identify potential risk factors, predict outcomes, and provide evidence-based guidance to assist surgeons in making informed decisions, adjusting their approach, and ensuring precision and accuracy in surgical interventions. This data-driven approach to surgical decision-making empowers healthcare providers to proactively manage risks, optimize outcomes, and prioritize patient safety in spine surgeries [13–16].

Furthermore, machine learning algorithms can assist in real-time monitoring and predictive modeling during spine surgeries to identify deviations from the expected course, alert surgeons to potential complications, and provide timely interventions to mitigate risks and optimize patient safety. By leveraging predictive analytics and risk stratification models, machine learning technologies can help healthcare providers identify patients at higher risk of complications, tailor treatment plans to individual patient profiles, and implement targeted interventions to enhance patient safety and improve outcomes in spine surgeries. This proactive approach to patient safety in spine surgeries enables healthcare providers to anticipate and address potential risks, optimize care delivery, and ensure the highest quality of care for patients undergoing surgical procedures [17, 18].

Moreover, the integration of machine learning technologies into clinical practice can facilitate continuous learning, improvement, and innovation in spine surgery practices by analyzing outcomes data, identifying best practices, and optimizing care pathways based on real-world evidence. By leveraging machine learning algorithms to analyze and learn from clinical data, healthcare providers can enhance quality assurance, performance monitoring, and outcome measurement in spine surgeries, leading to improved patient safety, reduced variability in care delivery, and enhanced overall quality of care. This data-driven approach to quality improvement and innovation empowers healthcare providers to continuously optimize care delivery, enhance patient safety, and drive advancements in spine surgery practices through evidence-based decision-making and continuous learning [19–21].

While the potential benefits of leveraging machine learning for enhanced patient safety in spine surgeries are substantial, there are challenges and considerations that need to be addressed. Ethical concerns, data privacy issues, algorithm transparency, and bias mitigation must be carefully managed to ensure the ethical and responsible use of machine learning in healthcare settings. Additionally, the integration of machine learning technologies into clinical practice requires ongoing training, education, and collaboration between healthcare professionals, data scientists, and technology experts to ensure effective utilization and seamless implementation of these technologies [18].

## Predictive analytics: a game-changer in improving surgical outcomes

Predictive analytics has emerged as a game-changer in the realm of healthcare, particularly in improving surgical outcomes. In the context of spine surgeries, where precision, risk mitigation, and patient safety are paramount, the application of predictive analytics offers a transformative approach to enhancing the quality of care and optimizing surgical interventions. By harnessing the power of data-driven insights and advanced algorithms, healthcare providers can proactively identify potential risks, predict outcomes, and personalize treatment strategies to improve surgical outcomes and patient satisfaction [22, 23].

Predictive analytics in spine surgeries offers a crucial advantage by utilizing historical data to detect patterns and anticipate potential complications before they manifest. Through the analysis of diverse preoperative and intraoperative data—such as patient demographics, medical history, imaging studies, and surgical parameters—healthcare providers can predict postoperative issues like infections, bleeding, or neurological deficits. This predictive ability empowers surgeons to proactively adjust their surgical strategies, take preventive actions, and optimize patient care to minimize risks and enhance outcomes [2, 24].

Additionally, predictive analytics plays a vital role in real-time decision-making during surgeries, providing surgeons with actionable insights and recommendations to guide their interventions. By examining real-time data streams, including intraoperative monitoring data, imaging feedback, and surgical parameters, predictive analytics aids surgeons in making informed decisions, identifying potential challenges, and adapting their approach to ensure optimal results. This real-time support enhances surgical precision, reduces errors, and ultimately elevates patient safety and satisfaction [7, 17].

Moreover, the personalized nature of predictive analytics allows for the customization of treatment plans to suit individual patient requirements and risk profiles. By evaluating a patient’s specific characteristics, medical history, genetic factors, and imaging data, predictive analytics enables healthcare providers to design tailored treatment strategies that address each patient’s unique needs. This personalized approach not only enhances patient safety but also improves surgical outcomes, diminishes complications, and boosts overall patient contentment [2, 20].

While the benefits of predictive analytics in enhancing surgical outcomes are substantial, certain challenges and considerations must be addressed. Ethical issues concerning data privacy, algorithm transparency, and bias mitigation need careful management to ensure the ethical and responsible utilization of predictive analytics in healthcare settings. Moreover, successful integration of predictive analytics into clinical practice necessitates collaboration among healthcare professionals, data scientists, and technology experts to ensure efficient implementation and utilization of this technology [6, 18].

## Personalized care through machine learning: transforming spine surgery practices

Personalized care through machine learning has the potential to revolutionize spine surgery practices by tailoring treatment strategies to meet the unique needs and characteristics of individual patients. In an era where precision medicine and patient-centered care are gaining prominence, the integration of machine learning algorithms in spine surgery holds promise for optimizing outcomes, enhancing patient safety, and improving overall quality of care. By leveraging advanced analytics, predictive modeling, and data-driven insights, healthcare providers can deliver personalized treatment plans that are customized to each patient’s specific medical history, risk profile, and anatomical considerations [13, 14, 23].

One of the key advantages of personalized care through machine learning in spine surgery is its ability to analyze vast amounts of patient data to identify patterns, trends, and correlations that can inform treatment decisions. By integrating data from various sources, such as electronic health records, imaging studies, genetic information, and patient-reported outcomes, machine learning algorithms can generate personalized treatment recommendations that take into account the unique characteristics of each patient. This personalized approach enables healthcare providers to tailor surgical interventions, rehabilitation plans, and follow-up care to meet the specific needs and preferences of individual patients, leading to improved outcomes and enhanced patient satisfaction [18, 19].

Furthermore, machine learning algorithms can assist in risk stratification and outcome prediction, allowing healthcare providers to proactively identify patients who may be at higher risk of complications or suboptimal outcomes. By analyzing patient data and identifying risk factors, machine learning algorithms can help healthcare providers develop personalized risk profiles for each patient, enabling them to implement targeted interventions, adjust treatment plans, and optimize care to minimize risks and improve outcomes. This predictive capability empowers healthcare providers to deliver proactive, personalized care that is tailored to each patient’s unique risk profile, ultimately enhancing patient safety and quality of care in spine surgery practices [7, 22].

Moreover, the real-time decision support provided by machine learning algorithms can guide surgeons during procedures, offering valuable insights, recommendations, and alerts to optimize surgical outcomes and mitigate risks. By analyzing real-time data streams, such as intraoperative monitoring data, surgical parameters, and imaging feedback, machine learning algorithms can assist surgeons in making informed decisions, adjusting their approach, and ensuring precision and accuracy in surgical interventions. This real-time guidance can enhance surgical outcomes, reduce errors, and improve patient safety, ultimately transforming spine surgery practices and elevating the standard of care for patients undergoing spinal procedures [13, 21].

## Mitigating risks in spine surgeries: the role of artificial intelligence

Enhancing patient safety, optimizing outcomes, and elevating the quality of care in spine surgeries necessitates effective risk mitigation strategies. The integration of artificial intelligence (AI) technologies in spine surgery shows great potential in bolstering risk management, identifying potential complications, and refining surgical decision-making to minimize risks and prioritize patient safety. Through the utilization of AI algorithms, machine learning models, and predictive analytics, healthcare providers can proactively pinpoint risk factors, customize treatment plans, and refine surgical approaches to mitigate risks and enhance outcomes in spine surgeries [17, 24, 25].

A primary advantage of AI in risk mitigation during spine surgeries lies in its capacity to sift through intricate datasets and unveil patterns, trends, and correlations that may elude human clinicians. By analyzing extensive patient data encompassing medical histories, imaging results, surgical details, and outcomes, AI algorithms can detect risk factors, forecast potential complications, and stratify patients based on their distinct risk profiles. This predictive ability equips healthcare providers to preemptively address risks, tailor treatments, and deploy targeted interventions to curtail complications and optimize results in spine surgeries [19, 21].

Additionally, AI technologies can aid in surgical planning and decision-making by furnishing real-time guidance, insights, and recommendations to surgeons throughout procedures. Through the analysis of intraoperative data, monitoring of vital signs, and provision of feedback on surgical parameters, AI algorithms can empower surgeons to make informed decisions, refine their approaches, and ensure precision and accuracy in surgical procedures. This real-time decision support can enhance surgical outcomes, diminish errors, and enhance patient safety by steering surgeons through intricate procedures and helping them navigate risks and challenges as they emerge [1, 6].

Furthermore, AI-driven risk prediction models can help healthcare providers pinpoint patients at heightened risk of complications or adverse events prior to surgery. By scrutinizing patient data, clinical indicators, and predictive markers, AI algorithms can craft personalized risk profiles for each patient, enabling healthcare providers to implement targeted interventions, adjust treatment strategies, and optimize care to mitigate risks and enhance outcomes. This predictive capability empowers healthcare providers to deliver personalized, proactive care tailored to each patient’s unique risk profile, ultimately advancing patient safety and care quality in spine surgeries [5, 23].

## Enhancing surgical decision-making with AI: a focus on patient safety in spine procedures

Improving patient safety, optimizing outcomes, and enhancing the quality of care in spine procedures heavily relies on leveraging artificial intelligence (AI) technologies to augment surgical decision-making [26–28]. The incorporation of AI algorithms, machine learning models, and predictive analytics into spine surgery practices provides invaluable insights, real-time guidance, and personalized recommendations to aid surgeons in making informed decisions, refining their approaches, and ensuring precision and accuracy in surgical interventions. Through the utilization of AI technologies, healthcare providers can refine surgical decision-making processes, mitigate risks, and prioritize patient safety to deliver top-tier care in spine procedures [6, 21, 23, 29].

A significant advantage of employing AI to enhance surgical decision-making in spine procedures is its capability to analyze and interpret intricate data streams, including intraoperative monitoring data, surgical parameters, imaging feedback, and patient-specific information. By processing and synthesizing this data in real time, AI algorithms can furnish surgeons with actionable insights, alerts, and recommendations to steer their decision-making during procedures. This real-time decision support empowers surgeons to navigate complex surgical scenarios, adapt their approaches, and optimize outcomes by leveraging AI-generated insights to enhance precision, accuracy, and safety in spine surgeries [1, 14, 30].

Moreover, AI technologies can aid in tailoring personalized surgical plans by scrutinizing patient-specific data, clinical parameters, and predictive factors to craft customized treatment strategies that enhance outcomes and minimize risks. Through the utilization of predictive modeling and risk stratification algorithms, AI can assist healthcare providers in identifying patients at higher risk of complications, customizing treatment plans to individual patient profiles, and implementing targeted interventions to bolster patient safety and enhance outcomes in spine procedures. This personalized approach to surgical decision-making equips healthcare providers to deliver patient-centered care that is tailored to each patient’s distinct needs, characteristics, and risk factors, ultimately enhancing safety and care quality in spine surgeries [17–19, 31].

Additionally, AI-powered decision support tools can enhance communication, collaboration, and coordination among multidisciplinary healthcare teams involved in spine procedures. By offering real-time insights, alerts, and recommendations to surgeons, anesthesiologists, nurses, and other healthcare professionals, AI technologies can facilitate seamless communication, streamline decision-making processes, and ensure coordinated care delivery throughout the surgical procedure. This collaborative approach to surgical decision-making with AI can bolster teamwork, optimize resource utilization, and enhance patient safety by fostering a culture of communication, collaboration, and shared decision-making among healthcare providers engaged in spine surgeries [20, 23, 32].

In summary, the emphasis on enhancing surgical decision-making with AI in spine procedures is crucial for prioritizing patient safety, optimizing outcomes, and enhancing the overall quality of care in spine surgery practices. By harnessing AI algorithms, machine learning models, and predictive analytics, healthcare providers can equip surgeons with real-time insights, personalized recommendations, and decision support tools to refine precision, accuracy, and safety in surgical interventions. As AI continues to progress and evolve, its integration into clinical practice holds vast potential for transforming surgical decision-making processes, elevating care standards, and ensuring the highest quality of healthcare delivery for patients undergoing spine procedures.

## Conclusions

the utilization of machine learning techniques to enhance patient safety in spine surgeries represents a transformative approach to improving surgical outcomes, mitigating risks, and optimizing care delivery in spine surgery practices. By harnessing the power of machine learning algorithms, predictive analytics, and real-time decision support tools, healthcare providers can proactively identify risk factors, personalize treatment plans, and optimize surgical interventions to minimize risks and maximize patient safety. The integration of machine learning technologies into clinical practice empowers surgeons with actionable insights, personalized recommendations, and data-driven guidance to make informed decisions, adjust their approach, and ensure precision and accuracy in surgical procedures. As machine learning continues to evolve and advance, its role in enhancing patient safety in spine surgeries holds immense potential for revolutionizing risk management strategies, elevating the standard of care, and transforming surgical decision-making processes to prioritize patient safety, optimize outcomes, and improve the overall quality of care in spine surgery practices.

## Data Availability

No datasets were generated or analysed during the current study.

## References

[1] Akosman I, Lovecchio F, Lyons K, Sarmiento JM, Lans A, Ghaedina H. The emerging role of artificial intelligence in adult spinal deformity. Seminars in Spine Surgery; 2022: Elsevier.

[2] Saravi B, Hassel F, Ülkümen S, Zink A, Shavlokhova V, Couillard-Despres S (2022). Artificial intelligence-driven prediction modeling and decision making in spine surgery using hybrid machine learning models. J Personalized Med.

[3] Stahel PF, Belk KW, McInnis SJ, Holland K, Nanz R, Beals J, Gosnell J, Ogundele O, Mastriani KS (2024). The Rothman Index predicts unplanned readmissions to intensive care associated with increased mortality and hospital length of stay: a propensity-matched cohort study. Patient Saf Surg.

[4] Kudo MS, Gomes de Souza VM, Estivallet CLN, de Amorim HA, Kim FJ, Leite KRM (2022). The value of artificial intelligence for detection and grading of prostate cancer in human prostatectomy specimens: a validation study. Patient Saf Surg.

[5] Mallow GM, Siyaji ZK, Galbusera F, Espinoza-Orías AA, Giers M, Lundberg H (2021). Intelligence-based spine care model: a new era of research and clinical decision-making.

[6] Feng Z, Yang H, Zhang X, Hai Y. The clinical application of artificial intelligence technology in spinal surgery. Med Rob. 2023; Nov 21;1.

[7] Ker J, Wang L, Rao J, Lim T (2017). Deep learning applications in medical image analysis. Ieee Access.

[8] Lee MS, Grabowski MM, Habboub G, Mroz TE (2020). The impact of artificial intelligence on quality and safety. Global Spine J.

[9] Katsuura Y, Colón LF, Perez AA, Albert TJ, Qureshi SA (2021). A primer on the use of artificial intelligence in spine surgery. Clin Spine Surg.

[10] DelSole EM, Keck WL, Patel AA (2022). The state of machine learning in spine surgery: a systematic review. Clin Spine Surg.

[11] Maki S, Furuya T, Inoue M, Shiga Y, Inage K, Eguchi Y (2024). Machine learning and deep learning in spinal Injury: a narrative review of algorithms in diagnosis and prognosis. J Clin Med.

[12] Pereverzev V, Kazmin A, Sazhnev M, Panteleev A, Kolesov S. Artificial intelligence for predicting various conditions in spine surgery: a systematic review. 2021. Vol. 27, no. 6. P. 813–820.

[13] Lalehzarian SP, Gowd AK, Liu JN (2021). Machine learning in orthopaedic surgery. World J Orthop.

[14] Karabacak M, Margetis K (2023). A machine learning-based Online Prediction Tool for Predicting Short-Term postoperative outcomes following spinal tumor resections. Cancers.

[15] Chen K, Zhai X, Sun K, Wang H, Yang C, Li M. A narrative review of machine learning as promising revolution in clinical practice of scoliosis. Annals Translational Med. 2021;9(1). 10.21037/atm-20-5495 PMC7859734 33553360

[16] Ellahham S, Ellahham N, Simsekler MCE (2020). Application of artificial intelligence in the health care safety context: opportunities and challenges. Am J Med Qual.

[17] Saravi B, Zink A, Ülkümen S, Couillard-Despres S, Hassel F, Lang G (2022). Performance of artificial intelligence-based algorithms to predict prolonged length of stay after lumbar decompression surgery. J Clin Med.

[18] Gianfrancesco MA, Tamang S, Yazdany J, Schmajuk G (2018). Potential biases in machine learning algorithms using electronic health record data. JAMA Intern Med.

[19] Schönnagel L, Caffard T, Vu-Han T-L, Zhu J, Nathoo I, Finos K (2024). Predicting postoperative outcomes in lumbar spinal fusion: development of a machine learning model. Spine J.

[20] Wilson JP Jr, Kumbhare D, Kandregula S, Oderhowho A, Guthikonda B, Hoang S. Proposed applications of machine learning to intraoperative neuromonitoring during spine surgeries. Neurosci Inf. 2023:100143.

[21] Choudhury A, Asan O (2020). Role of artificial intelligence in patient safety outcomes: systematic literature review. JMIR Med Inf.

[22] Romiyo P, Ding K, Dejam D, Franks A, Ng E, Preet K (2021). Systematic review and evaluation of predictive modeling algorithms in spinal surgeries. J Neurol Sci.

[23] Saravi B, Zink A, Ülkümen S, Couillard-Despres S, Lang G, Hassel F. Artificial intelligence-based analysis of associations between learning curve and clinical outcomes in endoscopic and microsurgical lumbar decompression surgery. Eur Spine J. 2023:1–11. 10.1007/s00586-023-08084-7 38156994

[24] Joshi RS, Haddad AF, Lau D, Ames CP (2019). Artificial intelligence for adult spinal deformity. Neurospine.

[25] Arad D, Rosenfeld A, Magnezi R (2023). Factors contributing to preventing operating room never events: a machine learning analysis. Patient Saf Surg.

[26] Lynn LA (2019). Artificial intelligence systems for complex decision-making in acute care medicine: a review. Patient Saf Surg.

[27] Zhou S, Zhou F, Sun Y, Chen X, Diao Y, Zhao Y. The application of artificial intelligence in spine surgery. Front Surg. 2022;9:885599. 10.3389/fsurg.2022.885599 PMC9403075 36034349

[28] Ren G, Yu K, Xie Z, Wang P, Zhang W, Huang Y (2022). Current applications of machine learning in spine: from clinical view. Global Spine J.

[29] Yagi M, Yamanouchi K, Fujita N, Funao H, Ebata S (2023). Revolutionizing spinal care: current applications and future directions of artificial intelligence and machine learning. J Clin Med.

[30] Benzakour A, Altsitzioglou P, Lemée JM, Ahmad A, Mavrogenis AF, Benzakour T (2023). Artificial intelligence in spine surgery. Int Orthop.

[31] Charles YP, Lamas V, Ntilikina Y. Artificial intelligence and treatment algorithms in spine surgery. Orthopaedics & Traumatology: Surgery & Research. 2023;109(1):103456. 10.1016/j.otsr.2022.103456 36302452

[32] Rasouli JJ, Shao J, Neifert S, Gibbs WN, Habboub G, Steinmetz MP (2021). Artificial intelligence and robotics in spine surgery. Global Spine J.

